# Convergent Strabismus and Its Treatment

**Published:** 1898-09

**Authors:** 


**Convergent Strabismus and its Treatment.** By EDWIN HOLTHOUSE. Pp. xi., 177. London: J. & A. Churchill. 1897.

Here is presented a careful and elaborate enquiry into the refractive and visual conditions occurring in 144 cases of convergent strabismus which were specially examined in the clinic of Mr. Lang, at the Royal London Ophthalmic Hospital. In 142 of the cases the refraction was hypermetropic, the remaining 2 being myopic. Among the hypermetropes, the squint was periodic in 7 cases, constant monolateral in 123, and alternating in 12. The periodic cases occurred in very young children and were easily cured by glasses. In 75 per cent. of the cases of constant monolateral strabismus the onset occurred under the age of 5 years, while in a considerable number hereditary tendency could be clearly traced. In this class the error of refraction was, as a rule, greater in the squinting than in the fixing eye, and there was a distinct connection between the amount of the hypermetropia and the degree of the squint. The squinting eyes were, as a rule, more amblyopic than the fixing eyes, and the author brings forward a considerable amount of evidence in favour of the theory that this defect is a cause and not an effect of the squint. The cases of alternating strabismus were characterised by an early age of onset, and a low degree of hypermetropia. The difference between the refraction and visual power of the eyes was also less marked than in monolateral cases.

With regard to treatment, 30.85 per cent. of the cases were cured by glasses and 61.70 per cent. improved. In 40.42 per cent. of the patients an operation was considered necessary, leaving 28.74 per cent. which, although not cured by glasses, were so much improved by wearing them that no further treatment was considered advisable.

On the whole, it cannot be said that this essay adds much to our knowledge of the subject treated. The statistics collected certainly emphasise the fact that, in spite of the close relationship between convergent strabismus and hypermetropia, with or without amblyopia, there is in most if not in all of the cases some other factor in the causation of the abnormality, but as to what this may be Mr. Holthouse does not bring forward any satisfactory evidence.

